# Pain neuroscience education and motor imagery‐based exercise protocol for patients with fibromyalgia: A randomized controlled trial

**DOI:** 10.1002/brb3.70013

**Published:** 2024-09-11

**Authors:** Selin Kircali, Öznur Özge Özcan, Mesut Karahan

**Affiliations:** ^1^ Neuroscience, Health Sciences Institute Üsküdar University Istanbul Turkey; ^2^ Electroneurophysiology, Vocational School of Health Sciences Üsküdar University Istanbul Turkey; ^3^ Medical Laboratory Techniques, Vocational School of Health Sciences Üsküdar University Istanbul Turkey

**Keywords:** fibromyalgia, motor imagery exercise, neuroscience, pain

## Abstract

**Background:**

This study is a randomized controlled, biopsychosocial study investigating the effectiveness of pain neuroscience education (PNE) and motor imagery‐based exercise protocol (MIEP) on fibromyalgia pain.

**Methods:**

Our study has four groups (MIEP *n* = 12, PNE *n* = 12, MIEP + PNE *n* = 14, Control *n* = 12) and all participants (*n* = 50) consist of patients diagnosed with fibromyalgia with chronic back pain. The primary outcome measure was pain intensity, and secondary outcome measures were beliefs, kinesiophobia, anxiety–depression, cognitive–mood, self‐esteem, and body awareness.

**Results:**

A statistically significant decrease in pain intensity was observed in all experimental groups, without any group being superior (Visual Analog Scale [VAS]: MIEP + PNE *p* = .003, 95% confidence interval [CI], −4.7078 to −0.9922; MIEP *p* = .003, 95% CI, −5.4806 to −1.0194; PNE *p* = .002, 95% CI, −3.6139 to −1.5461). There was a significant improvement in organic beliefs in both groups where PNE was applied (MIEP + PNE: *p* = .017, 95% CI, −7.8211 to −0.3189; PNE: *p* = .003, 95% CI, −9.7999 to −0.0401). A significant superiority in organic pain beliefs was detected in the MIEP + PNE group compared to the control group (*p* = .008, 95% CI, 1.7241–9.4959).

**Conclusions:**

According to this study, in which MIEP and PNE were combined, there was a decrease in pain intensity when both applications were applied together and when they were applied one by one. MIEP has improved her motor imagery ability, improved pain and increased body awareness. PNE has improved people's organic pain beliefs; removed people from fears, catastrophizing, and negative thoughts about pain; improved easier management of psychological processes and cognitive–emotion regulation ability.

## Graphical abstract

This study showed that the combined application of pain neuroscience education (PNE) and motor imagery‐based exercise protocol (MIEP) had a reducing effect, although not superior, on pain, which is the primary outcome, compared to individual applications In particular, PNE was superior in reducing cognitive–emotion regulation, anxiety, and depression, and MIEP was superior in reducing body awareness. No intergroup superiority was found on pain catastrophizing, self‐esteem, and kinesiophobia in the control and all intervention groups.

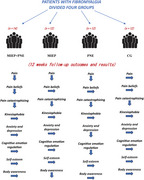

## 1 INTRODUCTION

As a result of the studies initiated by the International Association for the Study of Pain (IASP), according to the IASP council, the definition of pain is “an unpleasant sensory and emotional experience associated with actual and/or potential tissue damage” (Raja et al., 2020).

Fibromyalgia (FM) is a rheumatological disease characterized by widespread pain and tenderness. The presence of “chronic widespread pain” prevails without any tissue damage in the musculoskeletal system. Typical symptoms of FM are spontaneous pain in muscles and joints (allodynia), hyperalgesia, extreme thermal sensitivity, and extreme sensitivity to external stimuli such as chemicals, smells, sounds, and light. It also includes fatigue, sleep disturbance, morning stiffness, anxiety–depression, and cognitive disorders (Brum et al., 2022).

Pain is a highly individual, multidimensional, and complex process; whether or not it was formed through a comprehensive evaluation, taking into account all our experiences, thoughts, feelings, and beliefs. It has been proven that the “brain” definitely decides for its emergence and that it has a neurophysiological basis. When the balance of the loads on the nervous system is disrupted, alarm bells begin to ring in the system and the brain decides that the system is in danger and creates pain (Butler & Moseley, 2003). There are biological, psychological, and sociological burdens on our body, and all of these stresses are effective in the formation of pain. Accordingly, this study argues that pain should be addressed within the framework of the biopsychosocial model, as in current pain approaches.

Motor imagery (MI) is the mental execution of a movement without actually performing any movement and without stretching the muscles (Sengul et al., 2021). MI can be used to improve motor performance and learning motor tasks, inducing activation of various cortical areas, influencing the central nervous system, and causing changes in the brain (Grande‐Alonso et al., 2020). A neurocognitive rehabilitation approach with MI also improves pain recognition and perception in FM patients. It is effective in reducing pain and improving related symptoms (Paolucci et al., 2018).

Pain neuroscience education (PNE), as a relatively new and promising approach, is an educational content that aims to reconceptualize pain by explaining the neurobiology and neurophysiology of pain related to pain experiences to people with chronic pain, rather than focusing only on tissue pathology (Puentedura & Flynn, 2016). PNE is a cognitive‐based intervention implemented to increase participants' knowledge about pain and change their attitudes and beliefs regarding pain (Willaert et al., 2020). It uses neurophysiological information to teach patients that pain can be overprotective and completely real, even in the absence of tissue damage (Ceballos‐Laita et al., 2020). Depending on the time of administration, PNE can be seen as a protection that allows taking precautions in acute pain situations and as a treatment/rehabilitation training in chronic pain situations (Louw et al., 2011). It has also taken on a health education role, aiming to provide up‐to‐date information on neuroscientific advances in the field of pain (Galan‐Martin et al., 2019). The main aim of this study is to find out whether applying both PNE and motor imagery‐based exercise protocol (MIEP) will primarily reduce the pain of FM. These therapies could show an evidence of improvement in FM patients. However, there are no studies evaluating their effectiveness in combination.

## 2 METHODS

### 2.1 Study design

**FIGURE 1:**
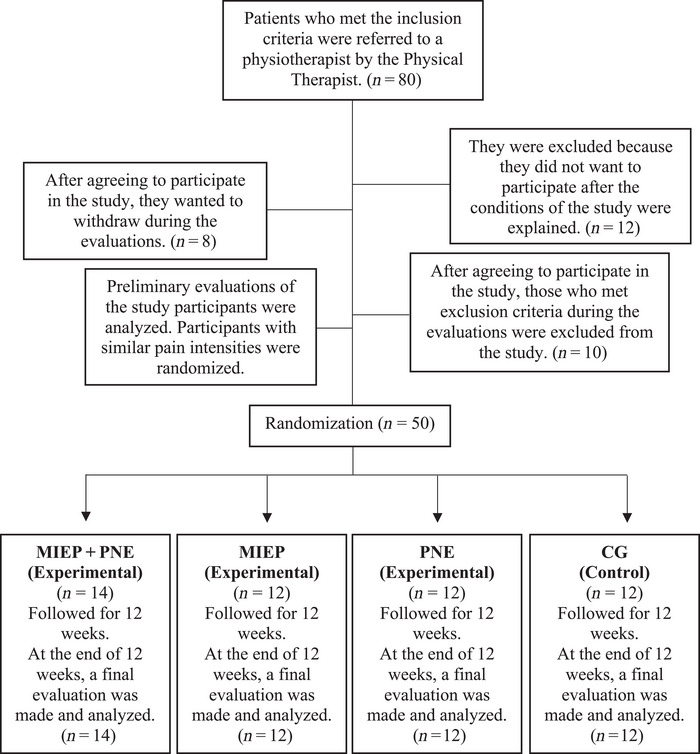
Flow chart of the study. CG, control group; MIEP, motor imagery‐based exercise protocol; PNE, pain neuroscience education.

This was a single‐center, prospective, assessor‐blinded, randomized controlled trial study. Ethics approval was obtained from the Clinical Research Ethics Committee of Uskudar University (approval number: E‐99102440‐/2022‐11). The study was registered at ClinicalTrials.gov (NCT05890326). Data collection was performed between November 2022 and May 2023. Moher et al.’s detailed randomized clinical trial guideline, the Consolidated Trial Reporting Standards (CONSORT) was considered for this study (Moher et al., 2012) and CONSORT flow chart in Figure 1 presents the research design. All participants were given all necessary and detailed information about the study procedure and consent forms were signed.

This study, which wanted to investigate the effectiveness of pain neuroscience training with MI exercises on chronic back pain, was designed as a four‐group study, including three experiments and a control group [(1) Exercise Protocol based on Motor Imagery (MIEP); (2) Pain Neuroscience Training (PNE); (3) Exercise Protocol based on Motor Imagery (MIEP) + Pain Neuroscience Training (PNE); (4) Control Group (CG)].

### 2.2 Participants, inclusion, and exclusion criteria

Baseline and follow‐up examinations were performed by an experienced, blinded physical therapist, and neurologist who referred patients who met the study criteria to the study physical therapist. The incoming patients were diagnosed with FM by a physical therapist and neurologist who are experts in the field, according to the protocol described by Wolfe et al. (2016). “The Fibromyalgia 2016 criteria require the following: (1) Widespread Pain Index (WPI) score ≥7 and Symptom Severity Score (SSS) score ≥5, or a WPI score 4–6 and SSS score ≥9; (2) the presence of widespread pain as defined above; and (3) symptoms of at least 3 months in duration (5).”

Conditions required to participate in the study were: (1) experiencing widespread chronic back pain for more than 12 months; (2) pain in at least 12 or more of 18 tender points with pressure of 5 kg/cm^2^; (3) ages 18–60; (4) not using pharmacological treatment; (5) not participating in any pain program; (6) not having participated in any physical exercise program in the last 2 years.

The conditions that constitute obstacles to participate in the study are (1) pregnancy; (2) currently continuing a physical exercise program; (3) the presence of a psychiatric disorder under psychological treatment; (4) the presence of major neurological and/or mental diseases such as Alzheimer's, dementia, and epilepsy; (5) the presence of physical and mental disabilities.

### 2.3 Procedure

Our study is a randomized controlled clinical study with four groups, one CG and three experimental groups. The experimental groups used “MIEP” and “PNE” applications both alone (2. group: MIEP, *n* = 12), (3. group: PNE; *n* = 12) as well as combined (1. group: MIEP + PNE; *n* = 14) there are three groups. There is also a fourth group that controls the experimental groups (4. group: CG; *n* = 12).

MIEP was performed twice a week (24 sessions in total) and PNE was performed once every 2 weeks (six sessions in total), with the entire sample (*n* = 50) being followed for 12 weeks.

### 2.4 Interventions: Motor imagery‐based exercise protocol

MIEP was referenced from Paolucci et al.’s (2022) previous study. Patients in the groups including MIEP received sessions of maximum 60 min in groups of three to four people, twice a week for 12 weeks. The MIEP intervention carried out in the study was blended and designed to best suit the patient group in the study by examining previous studies using a MI protocol. While selecting the exercises in the program, care was taken to ensure that the cervical–thoracic spine worked in every plane and axis, that the cervical–thoracic muscles were used, and that they included nerve mobilization. Before starting the study, video recordings of the designated exercises were made and a summary brochure about MI was prepared to give to the participants.

In the first session, the effects of MI were explained by giving a theoretical explanation, and at the beginning of each session, training on diaphragmatic breathing was given, as breathing exercises would be performed before MI. At the same time, at the end of each session, after MI, bodily awareness was stimulated by combining breathing and relaxation exercises. At the end of each session, people's opinions were received and their questions were answered. Relaxing meditation music was used in the background during MI and breathing exercises.

### 2.5 Interventions: Pain neuroscience education

PNE was applied face‐to‐face by PNE‐certificated researcher (Selin Kircali) referenced from Saracoglu et al. (2021). The training sessions were given as groups of five to six people, a maximum of 60 min and one in 2 weeks, for a total of six training sessions for 12 weeks. The PNE intervention performed in the study, as in similar studies in the literature, was mainly created by using the book “*Explain Pain*” written by David Butler and Lorimer Moseley for patients suffering from chronic pain; in addition, up‐to‐date information from current published articles on pain was also shared. Brochures summarizing the educational content were prepared before the start of the training sessions.

In each training session, people's opinions and thoughts were taken into account, interactive participation was ensured by allowing them to give examples on the subject, and people's questions were answered. Trainings were held primarily as face‐to‐face sessions, only held online if individuals were unable to attend face‐to‐face for any reason. The training sessions were organized and explained by the physiotherapist who was trained in pain neurophysiology and pain management, holds a master's degree in neuroscience, and also conducted the research. Although neurophysiological terms were used during the explanations, they were reduced to a simplicity that people could understand and metaphorical examples were used to make them memorable.

### 2.6 Control group

Participants in the CG did not receive any intervention and were followed for 12 weeks. Their final evaluation was carried out 12 weeks after the initial evaluation.

### 2.7 Randomization and blinding

Individuals who met the criteria had similar pain intensities, and agreed to volunteer for participation were given a volunteer code in the order of application. Without the knowledge of the patients, the papers with numbers from 1 to 50 were determined by drawing lots for the four groups through simple randomization. Whichever group and which numbers fall on it; people with that volunteer code were included in the group with that number.

Preliminary and final evaluations were made by the two physiotherapist (Selin Kircali and Öznur Özge Özcan) who managed the study and performed the applications and participated blindly in the recruitment of participants. The physical therapist (Öznur Özge Özcan) remained blind to the groups of patients she referred to as suitable for the study. The participants were also asked not to disclose any information about their treatment during the follow‐up assessments.

### 2.8 Outcome measurements

#### 2.8.1 Visual Pain Scale

Using a ruler, the score is determined by measuring the distance (mm) on the 10 cm line between the “no pain” anchor and the patient's mark, providing a score range of 0–10. A higher score indicates greater pain intensity. The Turkish validity study of the scale, the original version of which was developed by Freyd et al. in 2001, was conducted by Yaray et al. in 2011. As a result of the research, the Cronbach's alpha coefficient was 0.965, proving the validity of Visual Analog Scale (VAS) in Turkey (Yaray et al., 2011). Mease et al. (2011) reported that, in terms of clinical research on pain interventions in FM patients, these minimal clinical importance change differences (MCIDs) may correspond to decreases in scores of 32.3% and 34.2% from baseline.

#### 2.8.2 The Pain Beliefs Questionnaire (PBQ)

Beliefs about the causes and consequences of pain are collected in a total of 12 items, and eight of them (1, 2, 3, 5, 7, 8, 10, 11 substances) organic pain beliefs; four of them (4, 6, 9, 12 substances) express psychological pain beliefs. The evaluation is carried out with a six‐point Likert‐type scoring and the person marks one of the “always–almost always–often–sometimes–rarely–never” options according to the ratio of true/false items according to their own beliefs. The validity and reliability study of this scale in Turkey, which was first published by Edwards et al. in 1992, was conducted by Sertel et al. in 2006. As a result of the research, Cronbach alpha value for internal consistency was found to be 0.71 for organic beliefs and 0.73 for psychological beliefs, proving validity in Turkish (Sertel Berk, 2006). There is no reported reference MCID for Pain Beliefs Questionnaire (PBQ) in patients with FM.

#### 2.8.3 The Pain Catastrophizing Scale‐9

The Pain Catastrophizing Scale‐9 (PCS‐9) is a 13‐item self‐report measure designed to assess catastrophic thinking related to pain among adults with or without chronic pain. Patients are asked to rate the degree to which they have any of the thoughts described in the questionnaire using a five‐point Likert scale ranging from 0 (never) to 4 (always). Scores range from 0 to 52, with higher scores indicating a higher level of disaster. The validity and reliability study of the scale, which was first developed by Sullivan et al. in 1995, in Turkey was conducted by Uğurlu et al. in 2017. As a result of the research, Cronbach reliability coefficients for helplessness, rumination, magnification subscales, and total score were found to be 0.909, 0.856, 0.906, and 0.955, respectively, and validity and reliability were proven (Süren et al., 2014). The MCID for the PCS has been found to range from 3.2 to 4.5 in patients (Darnall et al., 2014).

#### 2.8.4 The Tampa Scale for Kinesiophobia

The Tampa Scale for Kinesiophobia (TSK) is a 17‐item questionnaire that quantifies fear of movement. Individual item scores range from 1 to 4, with the negatively worded items (4, 8, 12, 16) having a reverse scoring (4–1). The 17‐item TSK total scores range from 17 to 68 where the lowest 17 means no or negligible kinesiophobia, and the higher scores indicate an increasing degree of kinesiophobia. This scale, which was developed but not published by Miller et al. in 1991, was published by Vlaeyen et al. in 1995. The reliability study in Turkey was published by Tunca et al. in 2011. Test–retest reliability was applied in the study and the intraclass correlation coefficient (ICC) value was 0.806, resulting in excellent reliability (Tunca et al., 2011, 46). MCID for TSK was reported to be at least 4.5 points for patients with chronic musculoskeletal pain (Saracoglu et al., 2021).

#### 2.8.5 Hospital Anxiety and Depression Scale

In the 14‐item scale, seven evaluate anxiety (odd‐numbered items), seven evaluate depression (even‐numbered items), and scores between 0 and 7 are considered normal, scores between 8 and 10 are considered borderline, and 11 and above are considered abnormal. The Turkish validity and reliability study of the scale, which was first developed by Zigmond and Snaith in 1983, was conducted by Aydemir et al. in 1997, and the Cronbach's alpha coefficient was found to be 0.8525 for the anxiety subscale and 0.7784 for the depression subscale. Thus, the Turkish form was accepted as valid and safe (Aydemir et al., 1997). MCID for Hospital Anxiety and Depression Scale (HADS) was reported as 5.7 before (Longo et al., 2023).

#### 2.8.6 Cognitive Emotion Regulation Questionnaire

The scale, consisting of 36 items, measures under nine subheadings and these are “self‐blame, acceptance, focusing on negative thoughts, positive refocusing, refocusing on the plan, positive reconsideration, fixing the point of view, destruction, blaming others.” Evaluation is made with a five‐point Likert‐type scoring, and the person ticks one of the options “almost never–rarely–sometimes–often almost always” according to his/her own thoughts and feelings. The scale was first developed by Garnefski et al. in 2001, and its Turkish validity studies were conducted by Tuna and Bozo in 2012. As a result of the statistical analysis, the Cronbach alpha value was 0.90, revealing that Cognitive Emotion Regulation Questionnaire (CERQ) is a valid and reliable measurement tool (Tuna & Bozo, 2012). There is no reported reference MCID for CERQ in patients with FM.

#### 2.8.7 Rosenberg Self‐Esteem Scale

In the scale consisting of 10 items, participants choose one of the options “very true–true–wrong–very wrong” according to their thoughts. In his evaluation, the scoring options in the first five items were “4–3–2–1”. In the last five items, the order of the options is “1–2–3–4.” Between 30 and 40 points, a good level of self‐esteem. Between 26 and 29 points, a moderate level of self‐esteem. A score of 25 or less indicates low self‐esteem. The Turkish validity and reliability of the scale developed by Rosenberg in 1963 was proven by Çuhadaroğlu in 1986 (Tonga & Halisdemir, 2017). There is no reported reference MCID for Rosenberg Self‐Esteem Scale (RSS) in patients with FM.

#### 2.8.8 The Body Awareness Questionnaire

The Body Awareness Questionnaire (BAQ) is an 18‐item scale, with the total scale score calculated as a sum of the items. Items are scored on a 1–7 scale, with the total scale score calculated as a sum of the items. The questions with asterisks are reverse scored. The Turkish validity and reliability study of the questionnaire, which was developed by Shields and her friends in 1989, was conducted by Karaca in 2017. With the Cronbach's alpha value being 0.91, the Turkish version of BAQ was accepted as valid and reliable (Karaca, 2017). There is no reported reference MCID for BAQ in patients with FM.

### 2.9 Sample size

The number of samples was calculated with the G‐Power 3.1.9.4 program, taking into account the significance level and effect size of the established hypothesis. Based on the correlation coefficient between pain intensity and depression (*r* = 0.562, *p* < .05) obtained in Özer et al.’s study (2018), the high effect level was found to be 0.74 (effect size) (Özer et al., 2018). In order to find a significant difference in the study, when *α* = 0.05, 1 − *β* = 0.95, that is, the amount of error was 0.005 and the power of the test was 95%, the sample size was calculated as 44 people with a minimum of 11 in four groups (Özer et al., 2018).

### 2.10 Statistical analyses

Statistical analyzes “IBM SPSS Statistics for Windows.” It was performed using Version 25.0 (Statistical Package for the Social Sciences, IBM Corp.). Descriptive statistics are presented as *n* and % for categorical variables and as mean ± SD for continuous variables. When the data of the study were examined in terms of normality assumptions, Kolmogorov–Smirnov values were determined as *p* > .05. Wilcoxon test, one of the nonparametric tests, was applied to determine whether there was a significant difference between the scale and subscales before and after within the groups. Kruskal–Wallis test was used to compare the scale and subscales between groups. To determine which groups there was a significant difference, the Games–Howell test, one of the post hoc tests, was used. *p* < .05 was considered statistically significant.

## 3 RESULTS

In a four‐group study involving 50 participants, 14 participants received “MI exercises and pain neuroscience training” together in the combined group (Group 1: MIEP + PNE); 12 participants were in the group that received only “MI exercises” (Group 2: MIEP); 12 participants were distributed by simple randomization into the group that received only “PNE” (Group 3: PNE); and 12 participants into the CG without any application (Group 4: CG). The distribution of these participants' sociodemographic information such as gender, age, and education in the groups is shown in Table 1.

**TABLE 1:** Distribution of sociodemographic data about the participants.

Demographic variables	MIEP + PNE *n* = 14 (*n*, %)	MIEP *n* = 12 (*n*, %)	PNE *n* = 12 (*n*, %)	CG *n* = 12 (*n*, %)
Gender				
Woman	13 (92.9)	11 (91, 7)	12 (100, 0)	8 (66, 7)
Man	1 (7, 1)	1 (8, 3)	1 (0, 0)	4 (33, 3)
Age				
21–50‐years old	4 (28, 6)	8 (66, 7)	11 (91, 7)	3 (25, 0)
51–60‐years old	10 (71, 4)	4 (33, 3)	1 (8, 3)	9 (75, 0)
Education				
Primary school	2 (14, 3)	3 (25, 0)	0 (0, 0)	3 (25, 0)
High school	4 (28, 6)	0 (0, 0)	1 (8, 3)	6 (50, 0)
University	5 (35, 7)	7 (58, 3)	10 (83, 3)	2 (16, 7)
Master and above	3 (21, 4)	2 (16, 7)	1 (8, 3)	1 (8, 3)

Abbreviations: CG, control group; MIEP, motor imagery‐based exercise protocol; PNE, pain neuroscience education.

### 3.1 Outcome measurements

#### 3.1.1 Visual Pain Scale

No significant difference was detected in the preliminary VAS (pre‐VAS) scores of the participants in the groups, thus the groups consist of participants with similar VAS scores. There was no significant difference in the final VAS (post‐VAS) scores of the groups, so the groups do not have any superiority over each other in the applications (see Table 2).

**TABLE 2:** Comparison between groups.

Scales	Values		(1) MIEP + PNE (*n* = 14)	(2) MIEP (*n* = 12)	(3) PNE (*n* = 12)	(4) CG (*n* = 12)
**Pre‐VAS**	Mean ± SD		6.71 ± 1.94	7.17 ± 1.99	5.33 ± 0.89	5.33 ± 1.72
	*p* value	0.053				
	Post hoc	‐				
**Post‐VAS**	Mean ± SD		3.86 ± 2.77	3.92 ± 3.15	2.75 ± 1.48	5.25 ± 3.60
	*p* value	0.297				
	Post hoc	‐				
**Post‐PBQ**	Mean ± SD		23.64 ± 5.00	28.50 ± 5.16	23.75 ± 5.28	29.25 ± 4.52
**Organic beliefs**	*p* value	0.008*				
	Post hoc	**1–4** (*p* = .029)				
		1.72–9.49				
	95% CI	**(*p* < .05)**				
**Psychological beliefs**	Mean ± SD	0.274	18.14 ± 4.40	20.42 ± 3.48	20.33 ± 4.36	18.25 ± 4.73
	*p* value	‐				
	Post hoc					
**Post‐PCS‐9**	Mean ± SD		13.71 ± 6.18	18.17 ± 10.63	12.25 ± 8.07	15.17 ± 7.63
**Total**	*p* value	0.451				
	Post hoc	‐				
**Magnification**	Mean ± SD		2.86 ± 2.18	4.42 ± 2.68	3.92 ± 1.78	4.50 ± 2.15
	*p* value	0.174				
	Post hoc	‐				
**Rumination**	Mean ± SD		3.21 ± 1.97	5.00 ± 4.51	2.42 ± 3.29	4.08 ± 2.84
	*p* value	0.18				
	Post hoc	‐				
**Helplessness**	Mean ± SD		7.64 ± 2.90	8.75 ± 3.82	5.92 ± 3.92	6.58 ± 3.63
	*p* value	0.324				
	Post hoc	‐				
**Post‐TSK**	Mean ± SD		37.29 ± 4.75	38.83 ± 6.69	37.75 ± 4.69	43.42 ± 6.93
	*p* value	0.115				
	Post hoc	‐				
**Post‐HADS**	Mean ± SD		7.00 ± 3.31	9.17 ± 5.49	6.75 ± 3.49	8.00 ± 4.26
**Anxiety**	*p* value	0.753				
	Post hoc	‐				
**Depression**	Mean ± SD		4.14 ± 2.77	5.33 ± 3.92	4.58 ± 4.19	6.58 ± 4.12
	*p* value	0.35				
	Post hoc	‐				
**Post‐CERQ**	Mean ± SD		114.71 ± 10.49	117.67 ± 10.70	111.67 ± 8.81	114.25 ± 12.32
**Total**	*p* value	0.621				
	Post hoc	‐				
**Self‐blame**	Mean ± SD		11.21 ± 2.52	11.00 ± 2.95	11.17 ± 4.32	10.67 ± 2.06
	*p* value	0.963				
	Post hoc	‐				
						
**Acceptance**	Mean ± SD		12.21 ± 3.91	12.92 ± 2.27	11.92 ± 2.02	12.50 ± 3.00
	*p* value	0.82				
	Post hoc	‐				
**Focusing thought**	Mean ± SD		13.07 ± 1.69	14.58 ± 2.87	14.83 ± 1.85	12.58 ± 2.97
	*p* value	0.062				
	Post hoc	‐				
**Positive refocusing**						
	Mean ± SD		13.57 ± 1.91	13.50 ± 2.54	13.50 ± 2.47	13.83 ± 3.59
	*p* value	0.905				
**Refocusing on the plan**	Post hoc	‐				
	Mean ± SD		16.36 ± 2.59	15.92 ± 2.91	16.92 ± 2.19	16.00 ± 2.83
**Positive reconsideration**	*p* value	0.602				
	Post hoc	‐				
**Fixing the point of view**	Mean ± SD		15.29 ± 3.34	15.67 ± 2.46	15.75 ± 3.25	15.83 ± 3.59
	*p* value	0.977				
**Destruction**	Post hoc	‐				
	Mean ± SD		13.21 ± 3.09	14.25 ± 2.42	11.92 ± 3.00	13.67 ± 3.87
	*p* value	0.432				
**Blaming others**	Post hoc	‐				
	Mean ± SD		8.86 ± 2.85	10.58 ± 3.26	7.75 ± 2.67	8.83 ± 3.30
	*p* value	0.172				
	Post hoc	‐				
	Mean ± SD		10.93 ± 2.46	9.25 ± 2.67	7.92 ± 2.94	10.33 ± 3.14
	*p* value	0.066				
	Post hoc	‐				
**Post‐RSS**	Mean ± SD		26.43 ± 1.91	26.00 ± 2.30	27.33 ± 1.92	26.75 ± 2.49
	*p* value	0.503				
	Post hoc	‐				
**Post‐BAQ**	Mean ± SD		89.50 ± 13.47	102.58 ± 10.31	89.08 ± 7.84	98.50 ± 12.04
	*p* value	0.008*				
	Post hoc	2–3 (*p* = .08)				
		−21.2 to −5.7				
	95% CI					
**Post‐BAQ**	Mean ± SD					
	*p* value					
	Post hoc	1–2				
		(*p* = .46)				
	95% CI	2.92–23.2				
		**(*p* < .05)**				

KV = Kruskal–Wallis test; Post hoc = Games–Howell; *p < .05.

Abbreviations: BAQ, Body Awareness Questionnaire; CERQ, Cognitive Emotion Regulation Questionnaire; CG, control group; CI, confidence interval; HADS, Hospital Anxiety and Depression Scale; MIEP, motor imagery‐based exercise protocol; PBQ, Pain Beliefs Questionnaire; PCS‐9, Pain Catastrophizing Scale‐9; PNE, pain neuroscience education; RSS, Rosenberg Self‐Esteem Scale; SD, standard deviation; TSK, Tampa Scale for Kinesiophobia; VAS, Visual Analog Scale.

There was a statistically significant difference between pre‐ and post‐VAS scores in the MIEP + PNE (*p* = .003, 95% confidence interval [CI], −4.707 to −0.992), MIEP (*p* = .003, 95% CI, −5.480 to −1.019), and PNE (*p* = .002, 95% CI, −3.613 to −1.546) groups. The post‐VAS score was not statistically but clinically (>32.3% points) decreased in the interventions group, especially in MIEP group. VAS scores were found to be lower after than before. In the CG, no significant difference was found in the pre‐ and postresult measurements of VAS (see Table 3).

**TABLE 3:** Comparison of pre‐ and postoutcome measures.

Scales	Values	(1) MIEP + PNE (*n* = 14)	(2) MIEP (*n* = 12)	(3) PNE (*n* = 12)	(4) CG (*n* = 12)
VAS	Pre‐mean ± SD Post‐mean ± SD *p* 95% CI	6.71 ± 1.94 3.86 ± 2.77 .003* −4.70 to −0.99 **(*p* < .05)**	7.17 ± 1.99 3.92 ± 3.15 .003* −5.48 to −1.01 **(*p* < .05)**	5.33 ± .89 2.75 ± 1.48 .002* −3.61 to −1.54 **(*p* < .05)**	5.33 ± 1.72 5.25 ± 3.60 .953 −2.46 to 2.30 (*p* > .05)
PBQ Organic beliefs Psychological beliefs	Pre‐mean ± SD Post‐mean ± SD *p* 95% CI Pre‐mean ± SD Post‐mean ± SD p	27.71 ± 4.65 23.64 ± 5.00 .017* −7.82 to −0.31 **(*p* < .05)** 16.71 ± 5.00 18.14 ± 4.40 .431	30.33 ± 2.53 28.50 ± 5.16 .118 −5.27 to 1.61 (*p* > .05) 19.58 ± 3.75 20.42 ± 3.48 .504	28.67 ± 6.21 23.75 ± 5.28 .003* −9.79 to −0.04 **(*p* < .05)** 19.67 ± 1.92 20.33 ± 4.36 .195	30.50 ± 5.65 29.25 ± 4.52 .314 −5.58 to 3.08 (*p* > .05) 20.50 ± 4.03 18.25 ± 4.73 .141
PCS‐9 Total Magnification Rumination Helplessness	Pre‐mean ± SD Post‐mean ± SD *p* 95% CI Pre‐mean ± SD Post‐mean ± SD *p* Pre‐mean ± SD Post‐mean ± SD *p* 95% CI Pre‐mean ± SD Post‐mean ± SD *p* 95% CI	16.00 ± 5.70 13.71 ± 6.18 .314 3.50 ± 1.95 2.86 ± 2.18 .185 4.14 ± 2.80 3.21 ± 1.97 .376 8.36 ± 3.00 7.64 ± 2.90 .306	21.17 ± 13.27 18.17 ± 10.63 .433 5.33 ± 3.5 4.42 ± 2.68 .268 6.00 ± 4.37 5.00 ± 4.51 .474 9.83 ± 5.78 8.75 ± 3.87 .478	20.42 ± 9.31 12.25 ± 8.07 .006* −15.54 to −0.79 **(*p* < .05)** 5.25 ± 1.96 3.92 ± 1.78 .169 5.42 ± 3.45 2.42 ± 3.29 .007* −5.85 to −0.14 **(*p* < .05)** 9.75 ± 4.99 5.92 ± 3.92 0.025* −7.62 to −0.03 **(*p* < .05)**	17.92 ± 7.77 15.17 ± 7.63 .236 4.50 ± 2.07 4.50 ± 2.15 .832 4.58 ± 3.15 4.08 ± 2.84 .778 8.83 ± 3.66 8.58 ± 3.63 .902
TSK	Pre‐mean ± SD Post‐mean ± SD p	40.36 ± 4.63 37.29 ± 4.75 .068	38.83 ± 7.67 38.83 ± 6.69 .513	38.92 ± 5.53 37.75 ± 4.69 .449	42.58 ± 5.16 43.42 ± 6.93 .720
HADS Anxiety Depression	Pre‐mean ± SD Post‐mean ± SD *p* 95% CI Pre‐mean ± SD Post‐mean ± SD *p* 95% CI	8.36 ± 2.44 7.00 ± 3.31 .090 5.36 ± 2.84 4.14 ± 2.77 .066	9.75 ± 5.97 9.17 ± 5.49 .297 5.92 ± 3.20 5.33 ± 3.92 .229	8.50 ± 3.73 6.75 ± 3.49 .026* −4.80 to 1.30 6.42 ± 4.56 4.58 ± 4.19 .035* −5.54 to 1.86	7.50 ± 3.55 8.00 ± 4.26 .673 6.00 ± 3.69 6.58 ± 4.12 .469
CERQ Total Self‐blame Acceptance Focusing thought Positive refocusing Refocusing on the plan Positive reconsideration Fixing the point of view Destruction Blaming others	Pre‐mean ± SD Post‐mean ± SD *p* Pre‐mean ± SD Post‐mean ± SD *p* Pre‐mean ± SD Post‐mean ± SD *p* Pre‐mean ± SD Post‐mean ± SD *p* Pre‐mean ± SD Post‐mean ± SD *p* Pre‐mean ± SD Post‐mean ± SD *p* 95% CI Pre‐mean ± SD Post‐mean ± SD *p* 95% CI Pre‐mean ± SD Post‐mean ± SD *p* Pre‐mean ± SD Post‐mean ± SD *p* 95% CI Pre‐mean ± SD Post‐mean ± SD *p*	115.57 ± 13.33 114.71 ± 10.49 .972 10.93 ± 1.90 11.21 ± 2.52 .503 13.07 ± 2.53 12.21 ± 3.91 .179 12.43 ± 2.38 13.07 ± 1.69 .447 13.93 ± 2.64 13.57 ± 1.91 .580 16.43 ± 2.53 16.36 ± 2.59 .944 14.64 ± 3.10 15.29 ± 3.34 .574 13.43 ± 2.44 13.21 ± 3.09 .797 9.86 ± 3.63 8.86 ± 2.85 .529 10.86 ± 3.32 10.93 ± 2.46 .753	117.50 ± 13.17 117.67 ± 10.70 .636 11.33 ± 3.65 11.00 ± 2.95 .368 13.67 ± 2.83 12.92 ± 2.27 .395 15.00 ± 3.62 14.58 ± 2.87 .254 13.83 ± 2.48 13.50 ± 2.54 .726 15.58 ± 3.55 15.92 ± 2.91 .336 15.67 ± 3.17 15.67 ± 2.46 .932 12.92 ± 3.80 14.25 ± 2.42 .552 10.42 ± 4.14 10.58 ± 3.26 .959 9.08 ± 2.54 9.25 ± 2.67 .810	111.75 ± 8.18 111.67 ± 8.81 .937 11.50 ± 4.17 11.17 ± 4.32 .677 12.67 ± 2.19 11.92 ± 2.02 .500 15.33 ± 1.30 14.83 ± 1.85 .641 12.33 ± 2.02 13.50 ± 2.47 .088 15.42 ± 2.35 16.92 ± 2.19 .014* −0.42 to 3.42 **(*p* < .05)** 13.25 ± 3.39 15.75 ± 3.25 .005* −0.31 to 5.31 **(*p* < .05)** 13.25 ± 3.39 11.92 ± 3.00 .574 9.67 ± 2.27 7.75 ± 2.67 .007* −4.01to.17 **(*p* < .05)** 9.33 ± 2.99 7.92 ± 2.94 .151	107.58 ± 12.65 114.25 ± 12.32 .110 9.67 ± 3.37 10.67 ± 2.06 .237 11.33 ± 3.03 12.50 ± 3.00 .207 11.50 ± 2.88 12.58 ± 2.97 .263 14.00 ± 2.73 13.83 ± 3.59 .837 15.33 ± 2.64 16.00 ± 2.83 .230 15.50 ± 2.88 15.83 ± 3.59 .670 12.83 ± 2.72 13.67 ± 3.87 .206 8.17 ± 2.52 8.83 ± 3.30 .319 9.25 ± 2.90 8.83 ± 3.30 .173
RSS	Pre‐mean ± SD Post‐mean ± SD *p*	27.36 ± 1.95 26.43 ± 1.91 .059	26.67 ± 2.06 26.00 ± 2.29 .130	27.50 ± 1.88 27.33 ± 1.92 .615	25.58 ± 1.73 26.75 ± 2.49 .183
BAQ	Pre‐mean ± SD Post‐mean ± SD *p* 95% CI	88.71 ± 14.28 89.50 ± 13.47 .729	96.17 ± 15.44 102.58 ± 10.31 .041* −4.70 to 17.52 **(*p* < .05)**	92.92 ± 15.12 89.08 ± 7.84 .455	100.25 ± 13.92 98.50 ± 12.04 .556

*Z* = Wilcoxon signed ranks test, *p < .05 statistically significant.

Abbreviations: BAQ, Body Awareness Questionnaire; CERQ, Cognitive Emotion Regulation Questionnaire; CG, control group; CI, confidence interval; HADS, Hospital Anxiety and Depression Scale; MIEP, motor imagery‐based exercise protocol; PBQ, Pain Beliefs Questionnaire; PCS‐9, Pain Catastrophizing Scale‐9; PNE, pain neuroscience education; RSS, Rosenberg Self‐Esteem Scale; SD, standard deviation; TSK, Tampa Scale for Kinesiophobia; VAS, Visual Analog Scale.

#### 3.1.2 The Pain Beliefs Questionnaire

A statistically significant difference was found between the MIEP + PNE and CG groups in the scores in the final PBQ (post‐PBQ) organic beliefs subscale of the groups (*p* = .029, 95% CI, 1.724–9.495). The combined group was found to be superior to the CG (see Table 2).

A statistically significant difference was observed between pre‐ and post‐PBQ organic beliefs scores in the MIEP + PNE (*p* = .017, 95% CI, −7.821 to −0.318) and PNE (*p* = .003, 95% CI, −9.799 to −0.040) groups. The score was found to be lower after than before (see Table 3).

#### 3.1.3 The Pain Catastrophizing Scale‐9

No significant difference was detected in the final PCS‐9 (post‐PCS‐9) scores of the groups, thus the groups do not have any superiority over each other (see Table 2).

There is a statistically significant difference between pre‐ and post‐PCS‐9 in total (*p* = .006, 95% CI, −15.54 to −0.793), rumination (*p* = .007, 95% CI, −5.854 to −0.146), and helplessness (*p* = .025, 95% CI, −7.628 to −0.031) subscores in the PNE group. PCS‐9 total, rumination, and helplessness scores were found to be lower after than before (see Table 3) and clinically (>4.5 points) improved in the PNE group.

#### 3.1.4 The Tampa Scale for Kinesiophobia

No significant difference was detected in the final TSK (post‐TSK) scores of the groups, so the groups do not have any superiority over each other in the applications (see Table 2).

No significant difference was detected between pre‐ and post‐TSK scores. TSK scores were found to be lower after than before (see Table 3).

#### 3.1.5 Hospital Anxiety and Depression Scale

No significant difference was detected in the final HADS (post‐HADS) scores of the groups, so the groups do not have any superiority over each other in the applications (see Table 2).

Between pre‐ and post‐HADS, there was a statistically significant difference in the scores of the anxiety (*p* = .026, 95% CI, −4.808 to 1.308) and depression (*p* = .035, 95% CI, −5.547 to 1.867) subscales in the PNE group. HADS scores were found to be lower after than before (see Table 3) and not clinically (>5.7 points) improved in the intervention groups.

#### 3.1.6 Cognitive Emotion Regulation Questionnaire

No significant difference was detected in the final CERQ (post‐CERQ) scores of the groups, so the groups do not have any superiority over each other in the applications (see Table 2).

Between the pre‐ and post‐CERQ, the subscales refocusing on the plan (*p* = .014, 95% CI, −0.42 to 3.42), positive reconsideration (*p* = .005, 95% CI, −0.31 to 5.31), and destruction (*p* = .007, 95% CI, −4.01 to 0.17) showed a statistically significant difference in the PNE group. “Destruction” subscores were found to be lower after than before, and “refocus on the plan” and “positive reexamination” subscores were found to be higher after than before (see Table 3).

#### 3.1.7 Rosenberg Self‐Esteem Scale

No significant difference was detected in the final RSS (post‐RSS) scores of the groups, so the groups do not have any superiority over each other in the applications (see Table 2).

No significant difference was detected between pre‐ and post‐RSS scores. RSS scores were found to be lower after than before (see Table 3).

#### 3.1.8 The Body Awareness Questionnaire

A statistically significant difference was found between the final BAQ (post‐BAQ) scores of the groups. Post‐BAQ score was found to be higher in the MIEP group compared to the other groups (*p* = .008). As a result of the post hoc test, the MIEP group was statistically significantly superior to the PNE group (*p* = .008, 95% CI, −21.2 to −5.7) and the MIEP + PNE group (*p* = .046, 95% CI, 2.92–23.2; see Table 2).

There is a statistically significant difference between pre‐ and post‐BAQ scores in the MIEP group (*p* = .041, 95% CI, −4.70 to 17.52). BAQ scores were found to be higher after than before (see Table 3).

## 4 DISCUSSION

Our main purpose in this clinical study was to investigate the effectiveness of MIEP and PNE, especially on pain intensity. Additionally. The aim was to examine the effects of MIEP and PNE on pain, kinesiophobia, body awareness, psychological state, cognitive–emotional regulation state, and self‐esteem. According to the results of this study, PNE combined with MIEP treatment was associated with clinically significant improvement in pain and belief, while PNE and MIEP alone also resulted in clinical improvement during the 12‐week follow‐up period. Only MIEP application is more effective for body awareness and pain, PNE is superior for kinesiophobia, psychological state, cognitive–emotional regulation state, and self‐esteem according to pre‐ and postresults. Importantly, Saracoglu et al. (2021) reported 6‐week PNE sessions to pharmacological treatment was successful in improving functional status, pain, and level of kinesiophobia in patients with FM. However, there was no pharmacological intervention in our study. This study also showed that the inclusion of PNE was highly effective on kinesiophobia and pain in FM patients receiving pharmacological treatment, but in our results, the combination of MIEP and PNE did not show this effect.

Our study demonstrated that the combination of PNE and MIEP treatment was not resulted in superior outcomes in all scales in the 12‐week follow‐up period. The significant difference between baseline values in the intervention and CGs was felt not to be due to the randomization procedure because the baseline values of both groups were similar and not statistically different in primary outcome (pain) and other secondary outcome measures (all scales). This study has certain limitations. First, the participants were newly diagnosed patients who had not received any intervention before. This situation may have created extra tiring situations in the combined groups in terms of physical activity and therefore psychological effects. The number of participants was small so further studies are needed. MIEP improved motor visualization ability, improved pain, and increased body awareness. PNE enhanced people's organic pain beliefs; it distracted people from fears, disasters, and negative thoughts about pain. PNE and MIEP alone may contribute to psychological management of FM. Finally, these were the results of a 12‐week intervention in FM patients, and the long‐term effects of these interventions, either combined or alone, on patients are still unclear.

### 4.1 Motor imagery exercises

MI exercises are designed as a type of neuroscientific exercise to improve the virtual body map in the brain, and there are studies that show positive effects on chronic pain in current physiotherapy programs (Javdaneh et al., 2021). In a study conducted by MacIver et al. in 2008, it was observed that MI training provided a decrease in pain perception at the cortical level (Ribas et al., 2022). MI therapy used by Lindgreen et al. to cope with postsurgical pain has also yielded successful results. Vran et al. have shown that MI exercises in patients with chronic pain have potential benefits for the management of pain during painful activities (Sengul et al., 2021). Another clinical study suggested that stabilization exercises combined with MI exercises were superior to stabilization exercises alone in reducing pain, disability, and kinesiophobia in patients with chronic pain (Javdaneh et al., 2021). MI‐based rehabilitation has found a stronger effect on anxiety‐coping behavior in pain patients than traditional physiotherapy (Paolucci et al., 2022).

Studies in the literature on MI have shown that improvements in pain intensity, kinesthetic–visual imagery, fear of movement, anxiety–depression, cognitive status, and body awareness have been achieved through plasticity in the brain.

### 4.2 Pain neuroscience education

PNE redefines pain by eliminating fears and misconceptions about pain, creating a change in pain cognitions and perceptions. Thanks to this reconceptualization, participants can be more open to the activities and movements they feared before the training and can move away from catastrophizing thoughts about pain (Malfliet et al., 2018b). Thus, it allows changing cognition and erroneous beliefs, as well as improving functionality and physical condition (Galan‐Martin et al., 2019). Nijs et al. have supported PNE as a suitable method to prepare patients for cognition‐targeted exercise therapy. Many pain researchers, such as Moseley, Louw, Diener, Butler, Meeus, Ryan, Van Oosterwijck, and Puentedura, have suggested that pain neuroscience training programs provide positive improvements in improving pain intensity, pain knowledge of perceived disability, and pain cognitions, either alone or in combination with physiotherapy treatments (Orhan et al., 2021).

In studies conducted on PNE, positive results were obtained in people's pain intensity, beliefs about pain, catastrophizing thoughts about pain, fear of pain‐related movement, cognitive–emotional state, psychological process management, and body awareness after the training they received on pain.

There are a few studies in the literature investigating the effectiveness of MIEP on pain intensity, and these studies have shown that they are effective in reducing pain severity. In studies investigating the effect of PNE on pain severity, there were conclusions that pain severity was effective in addition to various physiotherapy applications, but that it was insufficient alone and further research was required. In this clinical study we conducted, our hypothesis was that both of these applications would show significant results on pain severity. The results showed a significant decrease in VAS scores between pre‐ and postmeasurements in all three experimental groups, supporting our hypothesis. As a result of the study, although pain intensity decreased in all groups, no significant superiority was detected between the groups. A decrease in pain intensity was observed both when MIEP and PNE were applied individually or together.

We expected participants' pain beliefs to change positively after PNE. There was a significant improvement in PBQ “organic beliefs” subscores in both groups where PNE was applied, and the MIEP + PNE group was found to be statistically significantly superior to the CG in outcome measurements. Thus, our hypothesis was confirmed and supported the studies in the literature.

After PNE, we expected that fear and bad thoughts about pain would decrease, as people with chronic pain would have a better perspective on pain management. Previous studies have also found that PNE provides a positive change in pain thoughts. According to PCS‐9 scores, significant results were found in the PNE group in terms of improvement in the total score, “rumination, which means repeated negative thoughts” and “helplessness” subscores. Although there was an improvement in scores in the MIEP + PNE combined group, no statistical significance was found. This may be due to the fact that the preliminary scores of the participants in the combined group regarding pain‐related fear and bad thoughts were not high; therefore, our hypothesis was partially proven. Although there was no significant superiority between the groups in the final measurements, PCS‐9 averages were seen to be lower in both groups where PNE was applied (lower only in the PNE group).

In studies where MIEP and PNE were investigated separately, it was observed that both of them made it easier for people to manage psychological processes. Our hypothesis in this study is that psychological process management will be better. According to the HADS scores, which are the two subscales of anxiety and depression, a significant improvement was observed in the PNE group in both subheadings between before and after measurements. Although no significant superiority was detected between the groups in the outcome measurements, the lowest score was seen in the groups where PNE was applied, and our hypothesis was partially supported.

While cognitive–emotional regulation abilities were expected to be better after PNE and MIEP, our hypothesis was partially proven by seeing a significant improvement in the CERQ subheadings of refocusing on the plan, positive reconsideration, and destruction “PNE only” group, and no significant superiority between the groups was detected in the outcome measurements.

Previous studies have shown that MIEP improves body awareness by improving visualization ability and perceptual virtual body map, and our hypothesis is that MI exercises have a positive effect on body awareness. Although an increase was observed in the BAQ score in both experimental groups where MIEP was applied, a significant improvement was seen in the “MIEP only” group. In addition, a significant superiority was detected in the MIEP group compared to the other experimental groups in outcome measurements. This result relatively supported the literature and confirmed our hypothesis.

Contrary to studies in the literature showing that MI reduces fear of movement due to pain, no significant results were obtained in any experimental group in our clinical study. Likewise, while an improvement was expected in self‐esteem, there was no significant change in RSS scores in any group. The reason for not getting the expected effect in these parameters may be that the participants in the study did not have a significant level of fear of movement on average in their first measurements, and similarly, they had average RSS scores in the first measurements.

## 5 CONCLUSION

According to this clinical study in which MIEP and PNE were combined, no superiority was detected between the groups in terms of pain intensity, and both applications are effective in reducing pain severity when applied together or individually.

MIEP improve MI ability, improve pain, and increase body awareness. When applied alone, MI exercises were found to be effective in improving body awareness, with significant superiority over other experimental groups.

PNE allows people to develop positively in their organic pain beliefs, distracts people from catastrophizing negative thoughts about pain, protects people from anxiety–depression by helping them manage psychological processes more easily, and improves a positive perspective in cognitive–emotion regulation. When applied alone, pain neuroscience training is effective in improving organic pain beliefs, pain thoughts, psychological processes and cognitive emotion regulation parameters.

Although there are more traditional physiotherapy practices in our country, specific therapy practices are mostly carried out in private rehabilitation centers. For example, MIEP techniques are performed by physiotherapists who are experts in their field, but this may increase the cost. As a result of the scarcity of PNE‐certified clinicians and their intense work, applying these treatments in combination and in groups on more patients can save time, reduce costs, and enable the application of more effective treatments on pain. Although the results we obtained from the study do not recommend combined treatment, they recommend that different studies be conducted on more FM patients. Finally, increasing the number of patients in future studies each group may contribute the generalize ability of our findings.

## AUTHOR CONTRIBUTIONS

**Selin Kircali**: Conceptualization; writing—original draft; investigation; validation; methodology; formal analysis. **Öznur Özge Özcan**: Writing—review and editing; writing—original draft; methodology; conceptualization; data curation; validation. **Mesut Karahan**: Conceptualization; supervision; project administration; writing—review and editing; methodology; writing—original draft; software; validation; visualization; funding acquisition; investigation; data curation; resources.

## FUNDING INFORMATION

This research received no specific grant from any funding.

## CONFLICT OF INTEREST STATEMENT

The authors declare no conflicts of interest.

### PEER REVIEW

The peer review history for this article is available at https://publons.com/publon/10.1002/brb3.70013.

## Data Availability

The data that support the findings of this study are available from the corresponding author upon reasonable request.
